# Statement on Antiplatelet Agents and Anticoagulants in Cardiology - 2019

**DOI:** 10.5935/abc.20190128

**Published:** 2019-07

**Authors:** Carlos V. Serrano Jr., Alexandre de M. Soeiro, Tatiana C. A. Torres Leal, Lucas C. Godoy, Bruno Biselli, Luiz Akira Hata, Eduardo B. Martins, Isabela C. K. Abud-Manta, Caio A. M. Tavares, Francisco Akira Malta Cardozo, Múcio Tavares de Oliveira Jr.

**Affiliations:** 1 Instituto do Coração do Hospital das Clínicas da Universidade de São Paulo, São Paulo, SP - Brazil; 2 Hospital Israelita Albert Einstein, São Paulo, SP - Brazil; 3 Hospital Beneficência Portuguesa Mirante, São Paulo, SP - Brazil

**Table:** 

Declaration of potential conflict of interest of authors/collaborators of the Statement on Antiplatelet Agents and Anticoagulants in Cardiology - 2019							
If the last three years the author/developer of the Statement:							
Names Members of the Statement	Participated in clinical studies and/or experimental trials supported by pharmaceutical or equipment related to the guideline in question	Has spoken at events or activities sponsored by industry related to the guideline in question	It was (is) advisory board member or director of a pharmaceutical or equipment	Committees participated in completion of research sponsored by industry	Personal or institutional aid received from industry	Produced scientific papers in journals sponsored by industry	It shares the industry
Alexandre de Matos Soeiro	No	Servier, Daiichi Sankyo	No	No	Sanofi	No	No
Bruno Biselli	No	No	No	No	No	No	No
Caio de Assis Moura Tavares	No	No	No	No	No	No	No
Carlos Vicente Serrano Júnior	No	No	No	No	No	No	No
Eduardo Bello Martins	No	No	No	No	No	No	No
Francisco Akira Malta Cardozo	No	No	No	No	No	No	No
Isabela C. K. Abud-Manta	No	No	No	No	No	No	No
Lucas Colombo Godoy	No	No	No	No	No	No	No
Luiz Akira Hata	No	No	No	No	No	No	No
Múcio Tavares de Oliveira Júnior	No	Boehringer Ingelheim, EMS	Sanofi Aventis, Boehringer Ingelheim, Roche Diagnostica, Philips Healthcare, Torrent Pharma	Torrent Pharma	Boehringer Ingelheim, Merck	EMS, Novartis, Torrent Pharma	No
Tatiana C. A. Torres Leal	No	No	No	No	No	No	No

## 1. Introduction

In 2013, the Brazilian Cardiology Society published the “Brazilian Guidelines on Antiplatelet and Anticoagulant Agents in Cardiology.” Over the past years, new studies have been carried out, providing important information on the use of these medications, administered alone and in combination with other medications. It is, therefore, time to review our guidelines and update them with this new knowledge which has been produced.

We have carried out an extensive review of the literature, and for this update we have chosen to emphasize 6 major topics in clinical practice which have undergone innovation over the past years or which were not covered in the previous document. The themes of this update are:

1. Antithrombotic therapy in patients using oral anticoagulants and undergoing percutaneous coronary intervention (PCI);
2. Duration of dual antiplatelet therapy following PCI;
3. Reversal of new anticoagulants;
4. Pericardioversion anticoagulation in atrial fibrillation (AF);
5. Anticoagulation and antiplatelet therapy in patients with patent foramen ovale;
6. Antithrombotic therapy in oncology patients with thrombocytopenia.

In this update, grade of recommendations and level of evidence were applied in accordance with the following standards.

It is our hope that this document may be of benefit to all professionals who, in their daily practice, face dilemmas and doubts regarding the best manner to prescribe various options and doses of available anticoagulants and antiplatelet agents.

**Table:** 

Grade of recommendation	
Grade I	Conditions for which there is conclusive evidence or, in the absence of conclusive evidence, general consensus that the procedure is safe and useful/effective
Grade IIa	Conditions for which there are conflicting evidence and/or divergent opinions regarding the procedure's safety and usefulness/effectiveness. Weight or evidence/opinion in favor of the procedure. The majority of studies/experts approve.
Grade IIb	Conditions for which there are conflicting evidence and/or divergent opinions regarding the procedure's safety and usefulness/effectiveness. Safety and usefulness/effectiveness less well established, with no prevailing opinions in favor.
Grade III	Conditions for which there is evidence and/or consensus that the procedure is not useful/effective and may, in some cases, be potentially harmful

**Table:** 

Level of evidence	
Level A	Data obtained from multiple concordant large randomized trials and/or robust meta-analysis of randomized clinical trials
Level B	Data obtained from less robust meta-analysis, from a single randomized trial, or from non-randomized (observational) trials
Level C	Data obtained through consensus of expert opinion

## 2. Antithrombotic Therapy in Patients Using Oral Anticoagulants and Undergoing Percutaneous Coronary Intervention

### 2.1. Introduction

Approximately 6% to 8% of patients undergoing PCI have a concomitant indication for long-term oral anticoagulant use, owing to various reasons such as AF, mechanical valves, or thromboembolism.^1-4^ It is, thus, fundamental to define the best way to treat these patients, especially regarding the combination of antiplatelet and anticoagulant medications.

For all patients who are receiving oral anticoagulants and who will undergo PCI, it is necessary to proceed to evaluating the need for maintaining anticoagulation and to calculate the risk of bleeding.

When AF is the reason for anticoagulation, the CHA_2_DS_2_-VASc score should be utilized, and maintenance should only be indicated when the score is ≥ 1 in men or ≥ 2 in women (Table 1). On the other hand, in patients with thromboembolic events or mechanical valve prostheses, anticoagulation should be maintained, regardless of any assessment.^5-7^

**Table 1:** CHA_2_DS_2_ VASc Criteria

	Description	Points
C	Congestive heart failure	1
H	Hypertension	1
A_2_	Age (≥ 75)	2
D	Diabetes mellitus	1
S_2_	Prior TIA or stroke	2
V	Vascular disease (prior AMI, PAD, or aortic plaque)	1
A	Age (65-74)	1
Sc	Sex (female)	1

AMI: acute myocardial infarction; PAD: peripheral arterial disease; TIA: transient ischemic attack.

Risk of bleeding should be assessed through the HAS-BLED score (Table 2). When it is ≥ 3, the patient is classified as at a high risk of bleeding. This should not contraindicate any form of treatment; however, it must be clear that the individual should be accompanied with more frequent consultations and that it is necessary to attempt to modify the risk factors present in the score in order to reduce the risk.^6-8^

**Table 2:** HAS-BLED Criteria

	Risk factor	Points
H	Arterial hypertension (SAP > 160 mmHg)	1
A	Abnormal kidney function: CrCl ≤ 50 mL/min or creatinine ≥ 2.26 mg/dL or hemodialysis or kidney transplant	1
	Abnormal liver function: bilirubin ≥ 2 × ULN or AST/ALT/AP ≥ 3 × ULN or hepatic cirrhosis	1
S	Prior stroke	1
B	Prior bleeding or predisposition to bleeding	1
L	Labile INR or < 60% time within therapeutic range	1
E	Age > 65	1
D	Drug use (NSAID, antiplatelet)	1
	Alcohol use (> 20 U per week)	1

ALT: alanine aminotransferase; AP: alkaline phosphatase; AST: aspartate aminotransferase; CrCl: creatinine clearance; INR: international normalized ratio; NSAID: nonsteroidal anti-inflammatory drugs; SAP: systolic arterial pressure; U: units; ULN: upper limit of normal.

### 2.2. Management of Antithrombotic Agents and the Moment of Percutaneous Coronary Intervention

Interrupting oral anticoagulation (OAC) during the periprocedural period can increase both the rate of bleeding and the rate of thromboembolic events.

Although there is no consistent evidence, the introduction of parenteral anticoagulants in patients receiving warfarin should only be considered when the international normalized ratio (INR) is less than 2.5. PCI may be performed while using anticoagulants; however, it should be postponed, if possible, until the patient’s INR is < 1.5, unless there is an emergency situation, and it is necessary to take high ischemia risk (GRACE score > 140, TIMI score ≥ 5, recurrent angina, refractory angina, hemodynamic instability, or ventricular arrhythmias) into consideration.^7,8^

Furthermore, for patients receiving new oral anticoagulants (NOAC), there is also no evidence as to whether parenteral anticoagulation or PCI should be performed early or not. Once more, when the risk of ischemia is very high, PCI should be performed early, while still under the effect of the medication. The procedure should be postponed, however, whenever the risk of ischemia permits. For patients with creatinine clearance > 50 mL/min, the full effect of NOAC may be considered reversed 24 hours after the last dose. For patients with creatinine clearance, on the other hand, between 30 and 50 mL/min, 48 hours are necessary. Following this period, the patient may thus theoretically undergo PCI with a lower risk of bleeding. Parenteral anticoagulation may be performed in the event that PCI is early, regardless of when the last dose of NOAC was administered.^7,8^

In all OAC patients, priority vascular access should always be radial, and femoral access should only be performed in exceptional cases.

The use of pre-PCI dual antiplatelet therapy should be routinely avoided in this group of patients. Clopidogrel should only be used once coronary anatomy has been defined and coronary angioplasty with stent placement has been indicated. The use of prasugrel or ticagrelor is contraindicated in this situation, as there is insufficient evidence for their safety in this context. Acetylsalicylic acid (ASA) should always be used at a minimum dose, preferably less than 100 mg daily.^7,8^

The use of proton pump inhibitors as prophylaxis against stress ulcers in this group of patients should be the first choice considered due to the elevated risk of gastrointestinal bleeding.^7,8^

### 2.3. Choosing Stent Type for Percutaneous Coronary Intervention

The choice of stent type (between the newest generation of drug-eluting stents and conventional stents) in patients who require full anticoagulation continues to generate discussion.

Results of the Dual Antiplatelet Therapy (DAPT) study (see section 2.2) showed that the benefits of prolonged dual antiplatelet therapy do not depend on the type of stent used and that the risk of coronary events in patients who suspend therapy due to the need for non-cardiac surgery was the same with either drug-eluting or conventional stents.^9-11^

Furthermore, two randomized studies have demonstrated that second-generation drug-eluting stents are superior to conventional stents in patients with high bleeding risks who were not able to tolerate the use of prolonged dual antiplatelet therapy.^12,13^

In this manner, stent choice should be individualized based on coronary anatomy and bleeding risk. There are, however, no reasons to contraindicate the use of drug-eluting stents in this group of patients.

### 2.4. Long-term Antithrombotic Therapy following Percutaneous Coronary Intervention

The first instances of evidence on this topic have begun to be published during the last five years, which means that the subject continues to be controversial and to produce doubts.

In 2012, data from the DANISH registry in patients with AF and acute myocardial infarction (AMI) showed that the 90-day risk of bleeding significantly increased with the use of triple therapy in comparison with anticoagulation combined with only one antiplatelet agent (hazard ratio [HR] = 1.47, 95% CI 1.04 to 2.08) with no differences in ischemic event rates (HR = 1.15, 95% CI 0.95 to 1.40). In this manner, analysis of this observational study would not recommend routine use of triple therapy.^14,15^

The What is the Optimal antiplatElet and anticoagulant therapy in patients with oral anticoagulation and coronary StenTing (WOEST) study, with 573 patients, was the first randomized prospective study published on this topic. All patients were indicated for OAC (69% for AF) and PCI. Patients were divided into 2 groups: warfarin and clopidogrel; and warfarin, clopidogrel, and ASA 80 mg daily. This treatment regimen was maintained for 30 days for conventional stents and 12 months for drug-eluting stents. The primary outcome was any bleeding episode according to TIMI criteria. After 1 one year, they observed a significant reduction in bleeding in the dual therapy group (19.5% versus 44.9%; HR = 0.36, 95% CI 0.26 to 0.50, p < 0.001). There was no different in rates of major bleeding, AMI, stent thrombosis, or stroke. Lower mortality, however, was observed in the dual therapy group (2.5% versus 6.4%, p = 0.027).^16^

In 2015, the Triple Therapy in Patients on Oral Anticoagulation after Drug Eluting Stent Implantation (ISAR-TRIPLE) multicenter randomized study, with 614 patients, conducted in Germany and Denmark, evaluated whether shortening the duration of clopidogrel therapy from 6 months to 6 weeks after drug-eluting stent implantation would be associated with superior net clinical outcome in patients receiving aspirin and warfarin concomitantly. They included patients who had been receiving oral anticoagulants for AF for at least 12 months and who had received a drug-eluting stent for stable angina or acute coronary syndrome (ACS). The primary outcomes were death, AMI, stent thrombosis, stroke, and major bleeding in 9 months. No differences were observed in relation to the primary outcomes between the 2 groups (9.8% versus 8.8%; HR = 1.14, 95% CI 0.68 to 1.91; p = 0.63). On the other hand, the incidence of minor bleeding events was higher in the group that used clopidogrel for 6 months (10.9% versus 7.3%, p = 0.03).^17^

With respect to NOAC, the PIONEER AF-PCI randomized prospective study evaluated the best pharmacological treatment strategy using rivaroxaban in patients who required OAC due to AF and who were undergoing PCI. The study included 2,124 patients, divided into 3 groups: rivaroxaban (15 mg) + P2Y_12_ inhibitor for 12 months; rivaroxaban (2.5 mg twice daily) + ASA + P2Y_12_ inhibitor for 1, 6, and 12 months; and warfarin + ASA + P2Y_12_ inhibitor for 1, 6, and 12 months. Approximately 93% of patients used clopidogrel as the antiplatelet of choice, and 65% received drug-eluting stent implantation. Approximately 50% of cases had ACS. The primary outcome evaluated was clinically relevant bleeding according to TIMI criteria. They observed bleeding rates of 16.8%, 18.0%, and 26.7%, respectively between the groups (p < 0.001). The rates of mortality, stroke, and cardiovascular events did not show any significant differences. For patients with AF who need stent angioplasty, the authors concluded that dual therapy or triple therapy strategies with reduced doses of rivaroxaban were safer and that they reduced bleeding rates in comparison with conventional triple therapy.^18^

Similarly, in 2017, the Evaluation of Dual Therapy with Dabigatran vs. Triple Therapy with Warfarin in Patients with AF that Undergo a PCI with Stenting (REDUAL-PCI) multicenter randomized prospective study included 2,725 patients with AF who underwent PCI, divided into triple therapy (warfarin + clopidogrel/ticagrelor + ASA) versus dabigatran + clopidogrel/ticagrelor. The primary outcome was major or clinically relevant bleeding. Furthermore, they tested the noninferiority of dual therapy in relation to thromboembolic events, death, and revascularization. The bleeding rate was 15.4% in the group that received 110 mg of dabigratan compared to 26.9% in the triple therapy group (p < 0.001 for noninferiority). In patients who received 150 mg of dabigratan, the bleeding rate was 20.2% compared to 25.7% in the triple therapy group (p < 0.001 for noninferiority). The combined events rates were 13.7% and 13.4% in the double and triple therapy groups, respectively (p = 0.005 for noninferiority). They mainly observed a reduced need for revascularization. The use of dual therapy with dabigatran was thus shown to be noninferior to triple therapy. As in the PIONEER AF-PCI study, the REDUAL-PCI study was not powered to show differences in coronary events or death. It was only possible to evaluate safety.^19^

**Figure 1:**
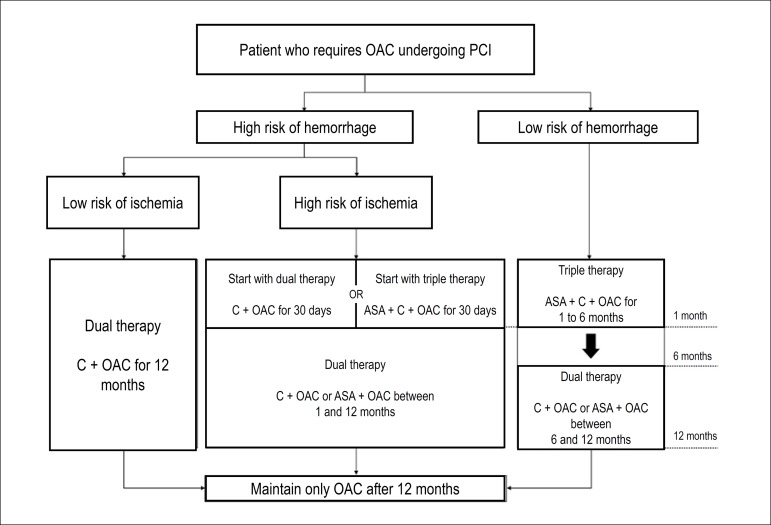
Flowchart representing indications for antithrombotic therapy combinations in accordance with ischemic and hemorrhagic risk. C: clopidogrel; OAC: oral anticoagulant; PCI: percutaneous coronary intervention.

Triple therapy should, thus, be considered only for patients with low hemorrhage risks during the shortest time possible (preferably 1 month, with the possibility of extending up to 6 months). After this period, anticoagulant use combined with just 1 antiplatelet agent should be maintained. However, when there is a high ischemia risk (Table 3) as well as a high hemorrhage risk, the recommendation is to use triple therapy for, at most, 1 month or to initiate dual therapy with an anticoagulant and clopidogrel directly (Table 4 and Figure 1).

**Table 3:** Definition of high long-term ischemic risk

Prior history of stent thrombosis during adequate antiplatelet therapy
"Final" artery angioplasty
Multiarterial coronary disease, especially in patients with diabetes
Chronic renal insufficiency (ClCr < 60 mL/min)
At least 3 stents and/or 3 lesions treated
PCI in bifurcation, with at least 2 stents placed
Total stent length > 60 mm
Treatment for chronic coronary occlusion

CrCl: creatinine clearance.

**Table 4:** Recommendation for managing patients who require oral anticoagulation undergoing percutaneous coronary intervention

Indications	Grade of recommendation	Level of evidence
The CHA_2_DS_2_-VASc score should be used to evaluate the need for maintaining anticoagulation, and the HAS-BLED score should be used to calculate the risk of bleeding	IIa	C
During PCI, priority vascular access should always be radial, and femoral access should only be performed in exceptional cases.	IIa	C
Triple therapy should be considered for the shortest time possible, due to the high associated risk of hemorrhage.	IIa	C
Utilization of NOAC should be given preference over warfarin, due to the predictability of their effect.	IIa	C
When opting to use warfarin, INR should be maintained close to 2.0.	IIa	C
Clopidogrel should only be used once coronary anatomy has been defined and coronary angioplasty with stent placement has been indicated, and routine pre-PCI administration should be avoided.	IIa	C
The use of prasugrel or ticagrelor is contraindicated in this situation.	III	C
ASA should always be used at a minimum dose, preferably ≤ 100 mg daily.	IIa	C
The use of proton pump inhibitors as prophylaxis against stress ulcers in this group of patients should be the first choice considered, due to the elevated risk of gastrointestinal bleeding.	IIa	C
Triple therapy should be considered for patients with low hemorrhage risks during the shortest time possible (preferably 1 month, with the possibility of extending up to 6 months). After this period, anticoagulant use in combination with just one antiplatelet agent should be maintained.	IIa	B
When there is a high ischemia risk as well as a high hemorrhage risk, the recommendation is to use triple therapy for, at most, 1 month or to begin dual therapy with an anticoagulant and clopidogrel directly.	IIa	B
In patients with high risks of bleeding and low ischemia risks, dual therapy with an anticoagulant and clopidogrel should be initiated from the beginning.	IIa	A
When opting to use NOAC, the combination of dual therapy with clopidogrel 75 mg daily and rivaroxaban 15 mg daily or dabigatran 110 mg twice daily should be the first choice.	IIb	B
Discontinuation of antiplatelet therapy should be considered after 12 months.	IIa	B

ASA: acetylsalicylic acid; INR: international normalized ratio; NOAC: new oral anticoagulant; PCI: percutaneous coronary intervention.

On the other hand, in patients with high risks of bleeding and low ischemia risks, in accordance with the results of the main published studies, the current recommendation is to initiate dual therapy with an anticoagulant and clopidogrel from the beginning (Table 4 and Figure 1).

It is, preferably, recommended to use NOAC instead of warfarin, due to the predictability of their effect. Furthermore, the NOAC should be chosen in accordance with the medical knowledge already established in this context and at doses previously studied in scientific research. When opting to use warfarin, the INR should be maintained close to 2.0.

Suspension of the antiplatelet agent is recommended 12 months after the last coronary event. Evidence for this conduct is scarce; it is, however, based on studies that showed that, after 1 year, anticoagulation is superior to ASA and that the combination, in addition to increasing bleeding rate, has no additional benefits.^20^ Combined therapy with clopidogrel and OAC may be prolonged for more than 1 year in patients with high ischemia risk and mechanical valve prostheses.

## 3. Duration of Dual Antiplatelet Therapy Following Percutaneous Coronary Intervention

### 3.1. Introduction

The combination of P2Y_12_ inhibitors with ASA monotherapy is known to be a great ally in the management of patients with coronary artery disease (CAD), be it acute or stable, and it reduces the risk of atherothrombotic phenomena, as well as stent thrombosis rates following PCI.^21-23^ This reduced ischemia risk is, however, indisputably associated with higher bleeding rates.^22-25^

The state of the art is to weigh the risks and benefits of dual therapy, as well as treatment duration, by contemplating clinical characteristics, the anatomical characteristics of the lesions addressed, and the type of stent used.

The risk of stent thrombosis in patients who have undergone PCI with conventional (metallic) stents is much more frequent during the first days and weeks following the procedure. In this manner, dual antiplatelet therapy is recommended for 1 month.^26^ With the advent of drug-eluting stents, conventional stents have been reserved, ideally, for patients with very high risks of bleeding who need shorter periods (at least 1 month) of dual antiplatelet therapy. In Brazil, however, conventional metallic stents continue to be used, especially in public health services.

Thrombosis in first-generation drug-eluting stents intensified perspectives on therapy duration in the past.^27^ Although the relative risk is still considerable, late or very late thrombosis in second-, third-, and fourth-generation drug-eluting stents has considerably reduced with modernization of the drugs eluted and the materials utilized. This has, thus, made it possible for dual therapy duration to be as short as possible, seeing that the risk of bleeding does not justify the small absolute benefit of reducing very late thrombosis. The use of first-generation drug-eluting stents is already infrequent in Brazil. For this reason, these guidelines do not include a discussion of treatment duration following percutaneous implantation of this type of stent.

The use of bioresorbable stents is already a reality in some centers. There are no studies to determine the ideal dual antiplatelet therapy duration specifically for this type of stent, although the recommendation for treatment duration is approximately 12 months. Some meta-analyses have suggested a lower rate of thrombosis in this type of stent in comparison with drug-eluting stents during the first 30 days following implantation. Use of the most potent P2Y_12_ inhibitors is thus indicated for this patient profile. There is still an increased risk of very late thrombosis, and longer treatment (more than 12 months) may consequently be considered for patients with low bleeding risks. Specific studies, however, are still lacking to reinforce this recommendation.^28-31^

### 3.2. Risk Scores

There are several known risk factors related to higher risks of ischemic events and bleeding episodes. Some of these factors are also related to both situations, which makes the medical decision even more complex.

Internationally implemented risk scores are currently available to aid in weighing treatment extension, taking the risk of bleeding and the benefit of reduced atherothrombotic risk into consideration.^32,33^ Based on studies, 2 scores have been elaborated: the DAPT score and the PRECISE-DAPT score.

The DAPT score (Table 5) was developed based on analysis of 11,648 patients included in the Dual Antiplatelet Therapy Study (DAPT) trial, and it has been validated in 8,136 patients who participated in the Patient Related Outcomes with Endeavor vs. Cypher Stenting (PROTECT) trial. Nine factors were identified: age, heart failure (HF) or reduced left ventricular ejection fraction (LVEF), saphenous vein graft stent implantation, AMI at initial presentation, prior AMI or prior PCI, paclitaxel-eluting stent, diabetes, stent diameter < 3 mm, and current tobacco use, resulting in a sum of points varying from −2 to +10. Patients with high DAPT scores (≥ 2) receive more benefits from prolonged dual antiplatelet therapy, as evaluated in cited study, over an average period of 30 months, given the reduction in AMI, stent thrombosis, and cardiovascular and cerebrovascular events, at the expense of a small increase in the risk of bleeding (NNT = 34 versus NNH = 272). On the other hand, patients with low DAPT scores (< 2) have an increased risk of events related to bleeding, with no reduction in the rate of cardiovascular and cerebrovascular events (NNH 64).^32^

**Table 5:** Factors used to calculate DAPT score. Scores ≥ 2 are associated with favorable risk-benefit, whereas scores < 2 are associated with unfavorable risk-benefit

Age ≥ 75	Points
Age > 65 and < 75	-2
Age < 65	-1
Current tobacco use	0
Diabetes mellitus	1
AMI at initial presentation	1
Prior PCI or AMI	1
Stent diameter < 3 mm	1
Paclitaxel-eluting stent	1
HF or reduced LVEF	1
Saphenous vein graft PCI	2
ICP em enxerto de veia safena	2

AMI: acute myocardial infarction; HF: heart failure; LVEF: left ventricular ejection fraction; PCI: percutaneous coronary intervention.

The Predicting bleeding complications in patients undergoing stent implantation and subsequent dual antiplatelet therapy (PRECISE-DAPT) included 14,963 patients undergoing elective, urgent, or emergency PCI, randomized into long (12 to 24 months) or short (3 to 6 months) dual antiplatelet therapy durations, with relation to bleeding risk based on 5 factors: age, creatinine clearance, hemoglobin, white-blood-cell count, or prior spontaneous bleeding (calculator available at www.precisedaptscore.com). In patients with high PRECISE-DAPT scores (≥ 25), prolonged therapy was associated with higher bleeding rates and no ischemic benefits; in contrast, patients with low PRECISE-DAPT scores had low combined adverse ischemic events with no significant increase in risk of bleeding.^33^

Scientific evidence tested in randomized studies is lacking to evaluate the real value of these scores in improving long-term outcomes for patients receiving dual antiplatelet therapy and undergoing PCI. Nevertheless, their utilization may be considered when individualizing decisions and contemplating the risks and benefits of prolonging dual antiplatelet therapy.

### 3.3. Dual Antiplatelet Therapy Duration following Percutaneous Coronary Intervention for Stable Coronary Artery Disease

Dual antiplatelet therapy in stable cases of CAD is not routinely indicated for patients receiving clinical treatment. It is only effectively necessary to indicate this following PCI, and the combination of ASA and clopidogrel is preferable.

There is no evidence from randomized studies on the use of prasugrel and ticagrelor as on option over clopidogrel for this patient profile. They are, however, options which may be considered for patients with high atherothrombotic risks if there is evidence that clopidogrel is not effective based on previous clinical outcomes or for patients who receive implantation of a bioresorbable stent.

Of the studies that evaluate dual antiplatelet therapy duration (ASA and clopidogrel) in stable patients, 3 are more recent, and they compare 6 months with 12 to 24 months of treatment. The Safety and Efficacy of 6 Months Dual Antiplatelet Therapy after Drug Eluting Stenting (ISAR-SAFE) study,^34^ which was the largest, randomized 4,005 patients and confirmed that there are no benefits and no reduction in ischemic events with the use of dual antiplatelet therapy for 12 months in comparison with 6 months. Similar results were also found in the Is There a Life for DES after Discontinuation of Clopidogrel? (ITALIC)^35^ and Second Generation Drug-eluting Stent Implantation followed by Six- versus Twelve-month Dual Antiplatelet Therapy (SECURITY) studies.^36^

Other meta-analyses^37,38^ have shown that 12 months of therapy did not add benefits in relation to reduced ischemic events in comparison with shorter therapy duration (< 6 months, including evaluation of studies that analyzed 3 months of therapy), which may be an option for patients with lower bleeding risks.

The DAPT trial and other meta-analyses^37-39^ have demonstrated that, in addition to reduced ischemic events, stent thrombosis, and infarction rates, as well as increased bleeding rates, therapy prolonged for more than 12 months showed a possible, albeit weak, relation to increased general mortality.

The recommendation of these guidelines is, thus, based on average dual antiplatelet therapy duration of 6 months following PCI in stable patients, with the possibility of considering a period of 3 months for patients with high bleeding risks. More prolonged use (> 12 months) is not routinely indicated and may be considered in accordance with the patient’s clinical and anatomical profile (Table 6).

**Table 6:** Recommendations on duration of dual antiplatelet therapy following percutaneous coronary intervention in stable coronary artery disease

Indications	Grade of recommendation	Level of evidence
In patients with stable CAD undergoing PCI who need dual antiplatelet therapy, the preferred combination is clopidogrel (75 mg) and a dose of ASA (75 to 200 mg)	I	A
The use of drug-eluting stents is always preferable to conventional stents, regardless of dual antiplatelet therapy duration	I	A
In patients with stable CAD undergoing PCI with a drug-eluting stent, dual antiplatelet therapy should be maintained for a minimum period of 6 months, regardless of stent type	I	A
In patients with stable CAD undergoing PCI with a conventional stent, dual antiplatelet therapy may be maintained for a minimum period of 1 month when there is a high risk of bleeding	I	A
In patients with stable CAD undergoing PCI with a drug-eluting stent who have high bleeding risks, the suspension of dual antiplatelet therapy may be considered	IIa	B
In patients with stable CAD undergoing PCI with a drug-eluting stent who tolerate the routine dual antiplatelet therapy duration without bleeding episodes and who have low bleeding risks and high atherothrombotic risks (DAPT score ≥ 2 and PRECISE-DAPT < 25), it is possible to maintain antiplatelet therapy for > 12 months and ≤ 30 months	IIb	A

ASA: acetylsalicylic acid; CAD: coronary artery disease; PCI: percutaneous coronary intervention.

### 3.4. Dual Antiplatelet Therapy Duration following Percutaneous Coronary Intervention for Acute Coronary Artery Disease

Ticagrelor and prasugrel are preferential P2Y_12_ inhibitors for patients undergoing PCI after ACS.

In patients who have ACS, the ischemia risk continues to be higher until approximately 1 year after the event, even after the culprit and non-culprit lesions have been treated.^22,20,40,41^ The main studies which affirm that ticagrelor and prasugrel have benefits over clopidogrel consider a reduction in events with an average treatment duration of 12 months.

A meta-analysis of 3 studies which compared 3, 6, and 12 months of dual therapy, including 11,473 patients, 4,758 of which had ACS, demonstrated that dual antiplatelet therapy for ≤ 6 months was associated with an increased risk of infarction, which was, however, not statistically significant. Considering the lower number of acute patients in comparison with studies that demonstrated the real benefits of intense dual antiplatelet therapy with ticagrelor and prasugrel (TRITON and PLATO), the discontinuation of dual antiplatelet therapy may be considered in patients with increased bleeding risks as early as 6 months after treatment initiation.^42^

The PEGASUS study evaluated patients who had had infarction 1 to 3 years prior to randomization. They studied the standard dose of ticagrelor (90 mg every 12 hours), a lower dose of ticagrelor (60 mg every 12 hours) and placebo. All patients received ASA and were followed for a median of 33 months. There was a significant reduction in the rates of infarction, cardiovascular death, and stroke, at the expense of an increase in events related to bleeding. The 60-mg dose of ticagrelor, however, demonstrated lower rates of bleeding in comparison with the 90-mg dose.^43^

In following with this, the recommendation of these guidelines is to administer dual antiplatelet therapy for at least 12 months to patients undergoing PCI after ACS, which may be modified to a minimum duration of 6 months in the event of increased risks of bleeding. In the same manner as in stable coronary disease, more prolonged use (> 12 months) is not routinely indicated and may be considered in accordance with patients’ clinical and anatomical profiles. When opting for this more prolonged treatment, a 60-mg dose of ticagrelor every 12 hours may be considered in combination with ASA (Table 7).

**Table 7:** Recommendations on duration of dual antiplatelet therapy following percutaneous coronary intervention in acute coronary syndrome

Indications	Grade of recommendation	Level of evidence
In patients with ACS undergoing PCI, regardless of stent type, dual antiplatelet therapy should be maintained for a minimum period of 12 months	I	A
In patients with ACS undergoing PCI with increased risks of bleeding, it is possible to consider maintaining dual antiplatelet therapy for a minimum period of 6 months	IIa	B
In patients with ACS undergoing PCI with a drug-eluting stent who tolerate the routine dual antiplatelet therapy duration without bleeding episodes and who have low bleeding risks and high atherothrombotic risks (DAPT score ≥ 2 and PRECISE-DAPT < 25), it is possible to maintain antiplatelet therapy for > 12 months and ≤ 30 months	IIb	A

ACS: acute coronary syndrome; PCI: percutaneous coronary intervention.

## 4. Reversal of New Anticoagulants

### 4.1. Introduction

At the same time that NOAC have been considered noninferior to vitamin K antagonists for ischemic stroke prevention in patients with AF and deep venous thrombosis treatment, they are related to lower risks of major bleeding, particularly hemorrhagic stroke.^44^

Furthermore, notwithstanding the current unavailability of antidotes for all NOAC, major bleeding episodes caused by these drugs do not seem to lead to worse clinical outcomes when compared to bleeding episodes in patients receiving vitamin K antagonists whose anticoagulant effects may be rapidly reversed.^45^

With the increased use of NOAC in clinical practice, the management of hemorrhagic events and the need to reverse anticoagulant effects in order to perform urgent procedures in patients receiving these medications have become common in emergency units.

In addition to general measures (Table 8), the utilization of antidotes, alternative therapies (e.g., prothrombin complex concentrates, factor VIIa, tranexamic acid) and the action of specialized teams (e.g., hematologists, endoscopists, neurologists, neurosurgeons, general surgeons, vascular surgeons, etc.) should be part of institutional protocols for reversing anticoagulation in the event of major bleeding episodes or urgent surgical procedures in patients receiving NOAC.^46^

**Table 8:** General measures for controlling major bleeding events in patients receiving NOAC

Mechanical compression when possible
Determining last dose of NOAC
Exams (renal and hepatic function, hemogram, complete coagulogram, and factor anti-Xa)
Volume expansion and red cell concentrate, when necessary
Activated charcoal if NOAC was taken < 2 hours prior

NOAC: new oral anticoagulant.

### 4.2. Antidotes

Three antidotes are in diverse developmental phases (Table 10). To date, only the monoclonal antibody idarucizumab has been approved for commercial use as an antidote for dabigratan. Andexanet alfa, an antidote for factor Xa inhibitors (rivaroxaban, apixaban, and edoxaban), has yet to be approved for commercial use in Brazil. Finally, ciraparantag, which is still in the early stages of development, is potentially capable of neutralizing the effects of both direct thrombin inhibitors and factor Xa inhibitors (Tables 9 and 10).

**Table 10:** Recommendations on the use of NOAC antidotes

Indications	Grade of recommendation	Level of evidence
The use of idarucizumab in patients receiving dabigatran is indicated at a dose of 5 g intravenous, when emergency intervention or surgery are necessary in patients who cannot wait the time it takes to metabolize the anticoagulant or in the event of life-threatening or uncontrollable bleeding episodes	IIa	B
In the event of surgeries or procedures which are elective or for which it is possible to wait the NOAC clearance time, controlled bleeding events, or anticoagulant overdoses without bleeding, the use of antidotes should not be indicated	III	C

NOAC: new oral anticoagulant.

**Table 9:** Reversal of NOAC anticoagulant effects8

NOAC	Specific antidote	Alternative therapeutic options
Dabigratan	Idarucizumab 5 g IV (divided in 2 doses of 2.5 g)	• PCC 50 IU/kg IV • RFVIIa 90 mcg/kg IV every 2 hours • Tranexamic acid 15 to 30 mg/kg IV • Hemodialysis
Rivaroxaban	Anti-factor Xa (e.g., Andexanet alfa - not approved)	• PCC 50 IU/kg IV • RFVIIa 90 mcg/kg IV every 2 hours • Tranexamic acid 15 to 30 mg/kg IV
Apixaban	Anti-factor Xa (e.g., Andexanet alfa - not approved)	• PCC 50 IU/kg IV • RFVIIa 90 mcg/kg IV every 2 hours • Tranexamic acid 15 to 30 mg/kg IV
Edoxaban	Anti-factor Xa (e.g., Andexanet alfa - not approved)	• PCC 50 IU/kg IV • RFVIIa 90 mcg/kg IV every 2 hours • Tranexamic acid 15 to 30mg/kg IV

IV: intravenous; NOAC: new oral anticoagulant; PCC: prothrombin complex concentrates; rFVIIa: recombinant activated factor VII.

Potential indications for the use of antidotes for NOAC include:

- A life-threatening bleeding episode (e.g. hemorrhagic stroke) or uncontrollable bleeding;
- Persistent bleeding, notwithstanding hemostatic measures;
- Risk of recurrent bleeding due to NOAC overdose or expected metabolism delay (e.g., renal insufficiency);
- Bleeding in non-compressible locations or in vital organs (e.g., retroperitoneal, pericardial, or intraocular bleeding or intramuscular bleeding with compartment syndrome);
- Need for emergency surgical intervention in patients with high risks of bleeding who cannot wait the time it takes to metabolize NOAC.

The use of antidotes does not seem to be necessary in patients who have received the last dose of NOAC more than 24 hours prior and who have creatinine clearance > 60 mL/min. In the event of elective surgeries or procedures, patients who may wait the time it takes to metabolize the NOAC, controlled bleeding, or anticoagulant overdose with no bleeding, antidote use does not need to be indicated.^47^

### 4.3. Idarucizumab

Idarucizumab is a monoclonal antibody fragment that neutralizes the anticoagulant effect of dabigatran by direct binding. Dabigatran, idarucizumab, and dabigatran-idarucizumab are eliminated by the kidneys.

The phase III Reversal Effects of Idarucizumab on Active Dabigatran (REVERSE-AD) study has shown that the intravenous use of 5 g of idarucizumab (2 consecutive doses of 2.5 g at 15-minute intervals) reverted the anticoagulant effect of dabigatran with normalization of thrombin time in more than 98% of patients, leading to early hemostasis in patients with major bleedings and low rates of hemorrhagic events in patients undergoing urgent surgery.^48^

#### 4.3.1. Andexanet alfa

Andexanet alfa is a recombinant factor Xa protein that binds to direct and indirect factor Xa inhibitors, removing them from circulation.

Phase II studies in healthy elderly patients have demonstrated that this drug, administered via intravenous bolus with subsequent continuous 2-hour infusion, reverted more than 90% of rivaroxaban and apixaban’s anti-Xa factor activity.^49^ The phase III Ability of Andexanet Alfa to Reverse the Anticoagulant Activity-4 study, which is currently underway, will evaluate the efficacy and safety of andexanet in controlling hemostasis in patients receiving rivaroxaban, apixaban, and edoxaban who have major bleedings. Interim analysis of the study with 67 patients showed a reduction in anti-Xa factor activity in 89% and 93% of patients using rivaroxaban and apixaban, with 70% clinical hemostasis.^50^

### 4.4. Alternative Therapies

Fresh frozen plasma (FFP), prothrombin complex concentrates (PCC), recombinant activated factor VII, and tranexamic acid are suggested as alternative therapies for patients receiving NOAC who have life-threatening hemorrhagic events or who need to undergo urgent procedures in the absence of a specific antidote (Table 9).^46^

Animal model, in vitro, and case series studies have demonstrated improved laboratory parameters for coagulation in patients receiving rivaroxaban.^51-53^ On the other hand, new evidence has suggested the superiority of recombinant activated factor VII and partially activated prothrombin complex concentrate (FEIBA) in relation to PCC in patients receiving rivaroxaban.^54,55^ A randomized placebo-controlled study in healthy individuals receiving dabigatran failed to show benefits of PCC use in improving laboratory parameters of coagulation.^56^ Furthermore, case series studies have shown controversial results for the use of FFP, PCC, recombinant activated factor VII, and fibrinogen.^57^

The absence of evidence on clinical reversal of anticoagulant effects of NOAC through the utilization of these alternative homeostatic agents, as well as conflicting data in relation to effects and optimal dosages, make routine use of these medications controversial.

Finally, hemodialysis may remove approximately 49% to 57% of circulating dabigatran in up to 4 hours, seeing that only 35% of the drug is bound to plasma proteins. Patients with renal insufficiency and dabigatran overdose may benefit from hemodialysis in the context of major hemorrhagic events or the need for urgent procedures (Table 10). As rivaroxaban and apixaban are highly bound to plasma proteins, they are not removed by hemodialysis.^46^

## 5. Pericardioversion Anticoagulation in Atrial Fibrillation

### 5.1. Introduction

AF is the most common sustained arrhythmia in clinical practice. Its incidence and prevalence increase with age, with a prevalence of 8% in the population over age 80.^58^ Furthermore, some American studies have shown that this prevalence increases by about 0.3% per year and that it had an absolute growth of 4.5% between 1997 and 2007.^58^ The reason behind this increase, in addition to population aging, is related to the improvements in treatment of chronic heart diseases, which increases the number of susceptible individuals, in addition to improved diagnostic tools with greater documentation of this arrhythmia.^59^

AF is associated with increased risk of stroke, as well as heart failure and total mortality.^60-64^ At least 20% of stroke cases are caused by AF, and these cases of stroke are generally more severe and incapacitating than ischemic stroke.^65-67^ Some studies have also shown increased risks of cognitive impairment secondary to asymptomatic embolic events in this population.^68^

Antithrombotic therapy plays a fundamental role in the prevention of embolic events when these risk factors are present, making it one of the main pillar of treatment, regardless of the strategy adopted (sinus rhythm or heart rate control).^69,70^ Risk may be calculated using the CHA_2_DS_2_-VASc score^71^ (Table 11), with an indication for chronic anticoagulation if the score is greater than or equal to 2 points, as long as there are no contraindications and the bleeding risk is acceptable.

**Table 11:** Recommendations on cardioversion anticoagulation in atrial fibrillation

Indications	Grade of recommendation	Level of evidence
Electrical cardioversion is recommended for patients with hemodynamic instability to reestablish cardiac output	I	B
Anticoagulation with heparin or a new oral anticoagulant should be initiated as soon as possible before any cardioversion for AF or flutter	IIa	B
In stable patients, with persistent AF, who are to undergo electrical or chemical cardioversion, OAC is recommended for at least 3 weeks before and 4 weeks after cardioversion within the therapeutic range (INR between 2 and 3). After 4 weeks, OAC maintenance should be in accordance with CHA_2_DS_2_VASc risk score	I	B
TEE is recommended to exclude thrombi as an alternative to periprocedural anticoagulation when early cardioversion is programmed	I	B
In the event that a thrombus is identified, anticoagulation should be maintained for 3 weeks	I	C
It is recommended to repeat TEE after 3 weeks of anticoagulation to ensure that the thrombus has been resolved before cardioversion	IIa	C
During the pericardioversion period, it is possible to opt for OAC with vitamin K antagonists or new anticoagulants, for the previously described duration	IIa	B
The use of OAC is indicated for patients with atrial flutter, with the same considerations as in AF	I	C
The preferred dose of rivaroxaban should be 20 mg daily, as long as there is a low risk of bleeding	I	B
The preferred dose of dabigatran should be 150 mg twice daily, as long as there is a low risk of bleeding	I	B
The preferred dose of apixaban should be 5 mg twice daily, as long as there is a low risk of bleeding	I	B
For patients undergoing electrical cardioversion guided by TEE without thrombi, UFH is recommended EV (bolus following by continuous infusion) before cardioversion and should be maintained until full OAC is reached	I	B
For patients with AF who need emergency electrical cardioversion, UFH is recommended EV (bolus following by continuous infusion)	I	C
For patients undergoing electrical cardioversion guided by TEE without thrombi, LMWH is recommended before cardioversion and should be maintained until full OAC is reached	I	B
For patients with AF who need emergency electrical cardioversion, a full dose of LMWH is recommended	I	C

AF: atrial fibrillation; EV: endovenous; INR: international normalized ratio; LMWH: low-molecular weight heparin; OAC: oral anticoagulation; TEE: transesophageal echocardiography; UFH: unfractionated heparin.

Warfarin is highly effective in preventing thromboembolic phenomena in AF, with a 64% reduction of risk in patients who receive adequate treatment.^72-74^ However, at least half of patients do not receive adequate treatment, for reasons that vary from difficulties in frequent INR monitoring to high risks of bleeding.^72,73^ Furthermore, patients receiving warfarin are not always within the appropriate therapeutic range (INR generally between 2 and 3), due to the occurrence of drug interactions (especially with antibiotic and anti-inflammatory drugs), food interactions, irregular medication use, and acute clinical intercurrences, among others.

With new anticoagulants becoming available over the past years, there have been improvements in relation to anticoagulation monitoring, given that they do not require INR monitoring, that they have few interactions with drugs and food, as well as elevated efficacy and safety, thus making it possible to increase treatment adherence and number of patients treated.^75^ The anticoagulants which have already been investigated are dabigatran, rivaroxaban, apixaban, and edoxaban. Dabigatran is a direct thrombin inhibitor, while the other 3 are factor Xa blockers, and they are used in clinical practice for patients with nonvalvular AF.

### 5.2. Strategies for Pericardioversion Anticoagulation in Atrial Fibrillation

When AF is reversed to sinus rhythm, either spontaneously or intentionally (via chemical or electrical cardioversion), the short-term risk of thromboembolism increases even more, with the majority of events occurring during the first 10 days after rhythm reversal.^76-81^ The group with the highest risk is that of patients with AF lasting more than 48 hours (1% to 5% during the first month, in the absence of anticoagulation).^82,83^

Embolism is a consequence of thrombus dislocation from the left atrium after the return of synchronous contraction; there may also, however, be thrombus formation after cardioversion, and this is the reason for indicating anticoagulation for at least 4 months following cardioversion, even in low-risk patients.^84-86^

The risk of thromboembolism may be reduced to 0% to 0.9% with anticoagulation for at least 3 weeks before cardioversion and 1 month after the procedure.^85-89^ A further option, with less anticoagulation time, is to evaluate the presence of atrial thrombi via transesophageal echocardiography (TEE) and, in the absence of thrombi, proceed to cardioversion, initiating full anticoagulation at the moment of the procedure and maintaining it for at least 4 weeks.

Warfarin is the most studied anticoagulant in this scenario;^90-99^ there is, however, sufficient evidence to use NOAC when performing the procedure, and NOAC are, moreover, preferable in some cases, for instance, when the patient is already receiving an NOAC, in order to shorten the precardioversion anticoagulation period (With warfarin, average time to adjust to adequate INR for at least 3 weeks is 6-8 weeks), or when there are difficulties in INR management.

Regarding pericardioversion, rivaroxaban has been compared to vitamin K antagonists in the X-VeRT study, which randomized 1,504 patients with AF of more than 48 hours or unknown duration to receive 1 of the 2 anticoagulants. There were no significant differences in the primary outcome, which was a composite of transient ischemic attack, stroke, peripheral embolism, AMI, and cardiovascular death (0.51% versus 1.02%; risk ratio [RR] 0.5; 95% CI 0.15 to 1.73), or the safety outcome, which was major bleeding (0.6% versus 0.8%; RR 0.76; 95% CI 0.21 to 2.67). Time to cardioversion was shorter in the rivaroxaban group.^79^ Furthermore, post hoc analysis of the ROCKET-AF study also showed no difference in events between patients who underwent cardioversion using rivaroxaban or warfarin (HR 1.38; 95% CI 0.61 to 3.11).^100^

With relation to dabigatran, which is a direct thrombin inhibitor, post-hoc analysis of the RE-LY study observed that 1,983 cardioversions were performed with 1,270 patients (from the total of more than 18,000 patients randomized in the original study). The incidence of stroke or systemic embolism in up to 30 days after cardioversion was similar between the groups receiving pericardioversion anticoagulation with warfarin, dabigatran 110 mg twice daily, and dabigatran 150 mg twice daily (0.6%, 0.8%, and 0.3%, respectively; p > 0.05). In the same manner, there were no differences in bleeding rates between the groups (0.6%, 1.7%, and 0.6%, respectively; p > 0.05). The results were not altered by prior TEE.77 Moreover, an observational Danish study, which compared 456 patients with nonvalvular AF receiving dabigatran with 774 patients receiving warfarin, provided evidence that there was a reduced median time to first cardioversion (4.0 weeks, with interquartile interval [IQI] of 2.9 to 6.5 compared to 6.9 weeks, with IQI of 3.9 to 12.1, for dabigatran and warfarin, respectively). No differences were observed between the 2 drugs regarding efficacy and safety.^101^

With relation to apixaban, post-hoc analysis of the ARISTOTLE study compared patients who underwent cardioversion receiving apixaban or warfarin. A total of 743 cardioversions occurred in 540 patients, with 265 patients in the apixaban group and 275 in the warfarin group. Average age (67 years old) and LVEF (around 52%) were similar in both groups, and average CHADS_2_ score in the apixaban group was 1.8 (± 1.0) and 1.9 (± 1.1; p = 0.17) in the warfarin group. There were no (zero) strokes or embolic events over 30 days in either of the groups, and 1 major bleeding episode occurred in each group.^78^ These data were subsequently confirmed in the EMANATE trial, which was presented at the European Congress of Cardiology in 2017 (to be published). In this study, 1,500 patients were randomized to receive apixaban or heparin/warfarin (conventional treatment) within 48 hours after cardioversion. Average age in both groups was approximately 64, and average CHA_2_DS_2_-VASc scores were 2.4 in both treatments. No ischemic events were observed in the apixaban group, while 6 events occurred in the conventional treatment group (p = 0.016). Finally, edoxaban was evaluated during the pericardioversion period in 2,199 patients in the ENSURE-AF randomized prospective study, in comparison with enoxaparin and warfarin, with enoxaparin being suspended when INR > 2. There were no differences regarding the primary outcome composed of stroke, systemic embolism, myocardial infarction, or cardiovascular death (odds ratio 0.46; 95% CI 0.12 to 1.4) or in the safety outcome of bleeding (odds ratio 1.48; 95% CI 0.64 to 3.55) for a total period of 28 days of treatment, followed up for more than 30 days.^80^

In specific situations, for instance, for patients who are highly symptomatic or patients with high bleeding risks, cardioversion may be performed earlier, without 3 weeks of anticoagulation, as long as there are no thrombi in the atria or atrial appendices evaluated by TEE. Subsequently, anticoagulation is maintained for at least 4 weeks.^71,102^ If TEE indicates that thrombi are present, anticoagulation is maintained for 3 weeks, and, in the event that cardioversion is scheduled, TEE should be repeated before the procedure. If there are doubts regarding medical adherence, TEE is also indicated in order to rule out thrombus.^78,103^

This strategy was first evaluated in a randomized manner by the ACUTE study, which compared TEE and the conventional strategy (anticoagulation for 3 weeks before cardioversion). Patients randomized to TEE received heparin if they were hospitalized or warfarin for 5 days before TEE if they were not hospitalized, and cardioversion was performed if there were no thrombi. If they had thrombi (12% of patients), cardioversion was postponed for 3 weeks, with anticoagulation maintained during this period. There were no differences between the TEE and conventional groups regarding the incidence of ischemic stroke (0.6% versus 0.3%, respectively, RR 1.95, 95% CI 0.36 to 10.60) or embolic events in general (0.8% versus 0.5%, respectively, RR 1.62, 95% CI 0.39 to 6.76) during the 8-week period following cardioversion. The majority of events in the TEE group occurred in patients whose AF recurred or who were outside of the therapeutic range at the moment of the event, whereas, in the patients who were receiving warfarin, the events occurred in sinus rhythm and within the therapeutic range. It is also interesting to note that fewer bleeding episodes were observed in the TEE-guided group (2.9% versus 5.5%; p = 0.03). In this group, time to cardioversion was also shorter (3.0 ± 5.6 days versus 30.6 ± 10.6 days; p < 0.001), and the success rate of AF reversal was higher in comparison with the conventional strategy (71% versus 65.2%; p = 0.03), although there was no difference in the percentage of patients who remained in sinus rhythm for 8 weeks.^104^

In the event that AF lasts less than 48 hours, which is easily determined by inquiring about symptoms, and the patient is not at a high thromboembolism risk (valve disease, ventricular dysfunction, prosthesis, prior history of thromboembolism), the risk of thromboembolism is very low and cardioversion may be performed^105,106^ without prior full anticoagulation. Maintaining anticoagulation for 4 weeks after the procedure is controversial and there are no studies comparing different heparins or heparins and new anticoagulants for patients with AF lasting less than 48 hours.

In the event of AF lasting less than 48 hours in patients with moderate to high risks of thromboembolic events (CHA_2_DS_2_-VASc > 1), unfractionated or low-molecular weight heparin before cardioversion and long-term maintenance are recommended.^86^

In cases of AF with hemodynamic instability, urgent cardioversion should always be performed, with a pre-procedure heparin bolus.^86^

In relation to heparins, unfractionated heparin has given way to low-molecular weight heparins. Indications for using heparins are: (1) following chemical or electrical cardioversion in hospitalized patients, (2) in combination with oral anticoagulation during INR adjustment; (3) during provisory interruption of warfarin anticoagulation in order to perform diagnostic procedures or therapies with hemorrhage risks (a strategy commonly known as a “heparin bridge”). Although there are 3 types of low-molecular weight heparin (dalteparin, enoxaparin, and nadroparin), enoxaparin has been the most widely used in clinical practice.^107^

**Figure 2:**
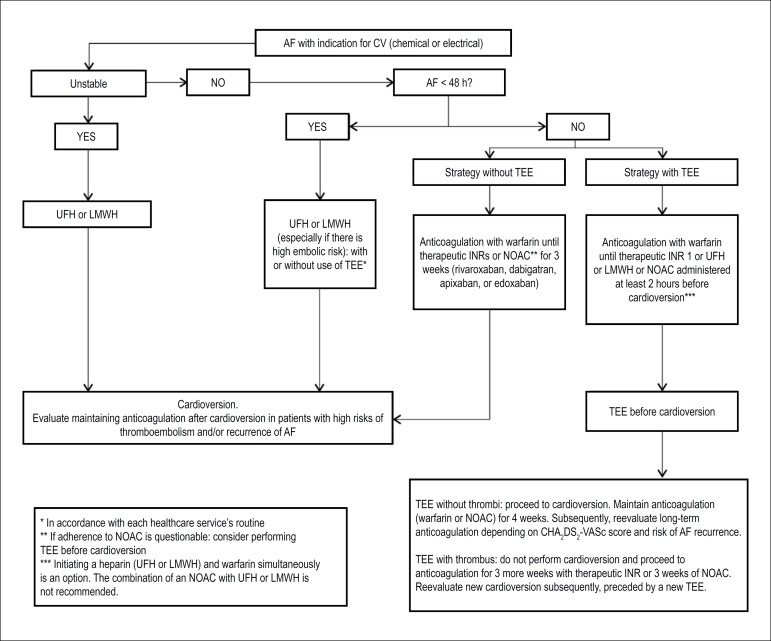
Recommendations in relation to pericardioversion anticoagulation in atrial fibrillation. AF: atrial fibrillation; INR: international normalized ratio; LMWH: low-molecular weight heparin; NOAC: new oral anticoagulant; TEE: transesophageal echocardiography; UFH: unfractionated heparin.

The flowchart in Figure 2 summarizes recommendations in relation to pericardioversion anticoagulation in atrial fibrillation.

## 6. Anticoagulation and Antiplatelet Therapy in Patients with Patent Foramen Ovale

### 6.1. Introduction

Patent foramen ovale (PFO) is the most common congenital heart disease of fetal origin.^108-110^ It is present in 15% to 35% of the adult population (15% to 25% in studies whose diagnostic method was echocardiogram^111,112^ and 15% to 35% in autopsies).^113-115^ Several reports exist on the relation between PFO and diverse pathologies, with different strengths of association, including: platypnea-orthodeoxia syndrome,^116^ decompression syndrome,^117,118^ systemic and coronary embolism,^119,120^ obstructive sleep apnea-hypopnea syndrome (OSAHS),^121^ migraine with aura,^122-125^ and stroke.^126^

### 6.2. Relation between Patent Foramen Ovale and Cryptogenic Stroke

The causal relation between PFO and cryptogenic stroke, caused by paradoxical embolism of the right-left shunt, is dubious and much debated within the literature.^112,127^ Meta-analysis data^128^ have established a possible causal relation between PFO and cryptogenic stroke in patients < age 55. Another study^129^ evaluated the presence of PFO using TEE in stroke patients and identified a higher incidence of PFO in cryptogenic stroke patients in comparison with patients with stroke of known cause, both in patients < age 55 and in patients > age 55. A retrospective Brazilian study^130^ also identified the presence of PFO as a risk factor for cryptogenic stroke, with an odds ratio of 3.3 in patients with PFO compared to patients without PFO. In prospective population studies, on the other hand, the presence of PFO has not been related to increased stroke risk, either in patients who have already suffered a stroke^131^ (secondary prevention) or in asymptomatic patients (primary prevention).^111,132,133^

The conclusion is, thus, that diagnosis of PFO in a patient with cryptogenic stroke does not establish a causal relation between the two entities,^134^ given that the risk attributable to PFO decreases with age and in the presence of risk factors such as systemic arterial hypertension, diabetes mellitus, tobacco use, personal history of transient ischemic attack, or prior stroke.^135^ Based on data from a meta-analysis^135^ of 12 cohort studies which followed cryptogenic stroke patients, the Risk of Paradoxical Embolism (RoPE) score, which quantifies the risk of PFO-attributable stroke (Table 12), was developed. This study provided evidence that patients with higher scores (with increased risks attributable to PFO as a causal factor of stroke) also had lower risks of stroke recurrence during follow up. In conclusion, the causal relation between PFO and stroke continues to be uncertain, and, even in patients whose stroke mechanism is attributed to PFO/paradoxical embolism, the event recurrence rate is very low (1% to 3% over 2 years in patients with RoPE scores of 9 or 10).^134,135^

**Table 12:** Risk of Paradoxical Embolism (RoPE) score. The higher the RoPE score, the higher the causality between patent foramen ovale and stroke

Characteristic	Points
No PH or SAH	1
No PH of diabetes	1
No PH of stroke/TIA	1
Non-smoker	1
Cortical infarct on imaging exam	1
Age (in years): 18 to 29 30 to 39 40 to 49 50 to 59 60 to 69 ≥ 70	5 4 3 2 1 0

PH: personal history; SAH: systemic arterial hypertension; TIA: transient ischemic attack.

Some echocardiography characteristics of PFO may be related to greater risks of paradoxical embolism, such as: significant right-left shunt, spontaneous right-left shunt, greater PFO flap mobility, prominent Eustachian valve, presence of Chiari network, and atrial septal aneurysm.^109,136-141^ However, some of these characteristics have not been shown to be consistently related to higher rates of embolic events in other studies.^111,132,142-145^

6.3. Evidence for the Use of Antiplatelet Agents or Anticoagulants in Patients with Patent Foramen Ovale

Given the hypothesis that embolic events related to PFO occur either by paradoxical embolism or embolism of a thrombus formed in the left atrium, antiplatelet therapy or anticoagulation are justified in the following situations:

Primary prevention: no studies have evaluated primary prevention of embolic events in patients with PFO. Considering that the causal relation between PFO and systemic embolism is still uncertain, that the embolic event rate in patients with PFO alone is extremely low, and that the risks inherent in anticoagulant and antiplatelet therapy are not negligible, the use of antiplatelet agents or anticoagulation are not indicated as primary prevention of embolic events in patients with PFO.^146^
Secondary prevention: the best therapeutic strategy after an embolic event in the presence of PFO continues to be the focus of debate and controversy due to the dubious correlation which exists between the 2 phenomena. In 2002, the PICSS substudy of the WARSS study^144^ compared the use of warfarin to ASA (325 mg daily) in patients with stroke and PFO, in a subgroup of 265 patients with cryptogenic stroke. There were no statistically significant differences in the rate of recurrent embolic events between the warfarin and the ASA groups in this situation. Another study^147^ randomized 47 patients after cryptogenic stroke to ASA (240 mg daily) or warfarin (with an INR goal of 2 to 3), and the authors did not observe any difference between the risk of ischemic stroke or TIA between the groups. A meta-analysis^148^ with data from only 2 randomized studies did not identify differences in favor of warfarin in comparison with ASA in the presence of stroke. Another recent meta-analysis^149^ compared the use of antiplatelet agents with oral anticoagulation in patients with cryptogenic stroke, using individual data of 2,385 patients from 12 observational studies, and they did not observe any differences in the rates of recurrent stroke between patients receiving oral anticoagulation or antiplatelet agents. In 2017, the results of the CLOSE study were published, which compared percutaneous PFO closure to medical therapy with antiplatelet therapy or anticoagulation. Statistical analysis was not conducted between the clinical treatment groups, as the study did not recruit the target of 900 patients and the event rate was lower than expected. When evaluating the data in absolute values, however, it is possible to observe an incidence of 3 cases in the anticoagulation group and 7 in the antiplatelet therapy group, with an estimated probability of stroke over 5 years of 1.5% and 3%, respectively.^45^ Percutaneous or surgical PFO closure should also be considered in select cases; this discussion, however, lies beyond the scope of this paper.

There is, thus, insufficient evidence for recommending the preferential use of oral anticoagulation over antiplatelet agents, given the low rate of recurrent embolic events in young patients with cryptogenic stroke. The use of antiplatelet agents, thus, seems to be adequate due to the accumulated risk of hemorrhagic complications in these patients in the event that they receive oral anticoagulation and to the efficacy of antiplatelet agents in reducing the risk of embolic events in the general population, which has already been proven (Table 13). It is worth highlighting that the studies on secondary prevention on which these guidelines are based^144,147,150^ were not designed to demonstrate the superiority of oral anticoagulation over antiplatelet therapy, for which reason they are not statistically powered to provide evidence of any possible benefits of oral anticoagulation over antiplatelet therapy.

**Table 13:** Recommendations for the use of antiplatelet agents and anticoagulants in primary and secondary prevention of cryptogenic stroke in patients with patent foramen ovale

Indications	Grade of recommendation	Level of evidence
Patients who are not indicated for anticoagulation for other reasons should be started on antiplatelet therapy as secondary prevention	I	B
Use of warfarin as a first choice following the first event	IIb	B
After a recurrent event while using antiplatelet agents, the use of warfarin with an INR goal between 2 and 3 should be considered	IIa	C
Use of Factor Xa inhibitors or thrombin inhibitors following the first event as an alternative to warfarin	IIb	C
Use of antiplatelet agents or anticoagulants as primary prevention	III	C

INR: international normalized ratio.

## 7. Antithrombotic Therapy in Oncology Patients with Thrombocytopenia

### 7.1. Introduction

Cardiovascular diseases and cancer are the main causes of death in Brazil.^151^ Advances in treatment of neoplasm have increased survival in this population which has thus gone on to be more exposed to traditional risk factors for developing atherosclerotic disease.

On the other hand, oncology patients, as they are in a pro-inflammatory and pro-thrombotic state, may develop atherosclerosis more quickly, and they have a higher risk of developing ACS.^152^

Neoplasia treatment with radiotherapy and chemotherapy itself has deleterious collateral coronary effects, such as the occurrence of vasospasms and endothelial injuries.^153^

Finally, the presence of thrombocytopenia increases the risk of both bleeding and ischemic phenomena. A retrospective evaluation at the MD Anderson Hospital showed that 39% of patients with ACS had platelet counts of < 100,000 cells/mm^3^.^154^

### 7.2. Antithrombotic Therapy

There are no randomized studies on antithrombotic therapy in patients with thrombocytopenia, as this population is normally excluded from large clinical trials.

A retrospective study of 70 oncology patients with ACS showed lower 7-day mortality in patients with thrombocytopenia who received ASA.^155^

In a case series which evaluated patients with platelet counts > 50,000 cells/mm^3^ who underwent angioplasty, the use of antiplatelet agents and anticoagulants did not increase the incidence of bleeding events.^156^

On the other hand, in patients with platelet counts between 30,000 and 50,000 cells/mm^3^, the use of ASA and clopidogrel was safe; lower doses of unfractionated heparin (30 IU/kg to 50 IU/kg), however, were enough to reach the therapeutic goal in this population.156 In patients with platelet counts below 10,000, risks and benefits should be evaluated individually. Platelet transfusion and antiplatelet therapy are a therapeutic possibility (Table 14).^156^

**Table 14:** Recommendations for use of antiplatelet agents and anticoagulants in oncology patients with thrombocytopenia

Indications	Grade of recommendation	Level of evidence
Use of acetylsalicylic acid in patients with coronary disease	I	A
Combined use of clopidogrel and acetylsalicylic acid in patients with high-risk acute coronary syndrome or after coronary angioplasty	I	A
Acetylsalicylic acid should always be used at a minimum dose, preferably ≤ 100 mg daily	IIa	C
Use of antiplatelet therapy and/or anticoagulant in acute coronary syndrome patients, even if they have thrombocytopenia	IIa	C
Use of a reduced dose of enoxaparin and unfractionated heparin in patients with platelet count < 50,000. Monitoring of therapeutic goal	IIa	C

There are no studies on new antiplatelet agents or non-vitamin K dependent anticoagulants in this population.
